# High HIV and syphilis prevalence among female sex workers and sexually exploited adolescents in Nimule town at the border of South Sudan and Uganda

**DOI:** 10.1371/journal.pone.0266795

**Published:** 2023-01-18

**Authors:** Alfred G. Okiria, Victoria Achut, Erin McKeever, Alex Bolo, Joel Katoro, Golda Caesar Arkangelo, Acaga Taban Ismail Michael, Avi J. Hakim

**Affiliations:** 1 IntraHealth International, Juba, South Sudan; 2 South Sudan Ministry of Health, Juba, South Sudan; 3 Division of Global HIV and TB, United States Centers for Disease Control and Prevention, Atlanta, GA, United States of America; 4 United States Centers for Disease Control and Prevention, Juba, South Sudan; Ohio University, UNITED STATES

## Abstract

HIV prevalence among the general population in South Sudan, the world’s newest country, is estimated at 2.9% and in Nimule, a town at the border with Uganda, it is estimated at 7.5%. However, there is limited data describing the HIV epidemic among female sex workers and sexually exploited adolescents (FSW/SEA) in the country. This study was conducted using a respondent-driven sampling (RDS) among FSW/SEA aged ≥15 years in January-February 2017 who sold or exchanged sex in the last six months in Nimule. Consenting participants were administered a questionnaire and tested for HIV according to the national algorithm. Syphilis testing was conducted using SD BIOLINE Syphilis 3.0 and Rapid Plasma Reagin for confirmation. Data were analyzed in SAS and RDS-Analyst and weighted results are presented. The 409 FSW/SEA participants with a median age of 28 years (IQR 23–35) and a median age of 23 years (IQR 18–28) when they entered the world of sex work, were enrolled in the Eagle survey. Nearly all (99.2%) FSW/SEA lacked comprehensive knowledge of HIV though almost half (48.5%) talked to a peer educator or outreach worker about HIV in the last 30 days. More than half (55.3%) were previously tested for HIV. Only 46.4% used a condom during their last vaginal or anal sexual act with a client. One in five (19.8%) FSW/SEA experienced a condom breaking during vaginal or anal sex in the last six months HIV prevalence was 24.0% (95% CI: 19.4–28.5) and 9.2% (95% CI: 6.5–11.9) had active syphilis. The multivariable analysis revealed the association between HIV and active syphilis (aOR: 6.99, 95% CI: 2.23–21.89). HIV and syphilis prevalence were higher among FSW/SEA in Nimule than the general population in the country and Nimule. Specifically, the HIV prevalence was eight times higher than the general population. Our findings underscore the importance of providing HIV and syphilis testing for FSW/SEA in conjunction with comprehensive combination prevention, including comprehensive HIV information, promotion of condom use, and availing treatment services for both HIV and syphilis.

## Introduction

Human Immunodeficiency Virus (HIV) infection rate is of public health importance in South Sudan, the world’s newest nation. The HIV epidemic in South Sudan is generalized, with variations in prevalence and disease burden by age, population, and location [1]. In 2018, the Joint United Nations Programme on HIV and AIDS (UNAIDS) Spectrum model estimated an adult (15–49 years) HIV prevalence of 2.5%, ranging from 2.0% amongst adult males to 3.0% amongst adult females [2]. According to the 2017 Antenatal Care (ANC) Sentinel Surveillance Survey, HIV and syphilis prevalence amongst women attending ANC were 2.9% and 8.1%, respectively. The prevalence of HIV was highest in the Western, Central, and Eastern Equatoria States, at 4.8%, 4.6%, and 3.6%, respectively [1]. HIV prevalence in Nimule was estimated to be 7.5% in the general population [1].

Key populations in South Sudan, including female sex workers and sexually exploited adolescents (FSW/SEA), are disproportionately affected by HIV. Little is known, and limited data exist, about HIV infection among FSW/SEA in South Sudan. A 2016 study involving 850 FSW/SEA participants in Juba found an HIV prevalence of 38%, with most of the FSW/SEA coming from the neighboring countries of Uganda, Kenya, and the Democratic Republic of Congo [3]. Even in generalized epidemics, FSW/SEA are affected due to behavioral, social, and structural factors [4–6]. The FSW/SEA are at increased risk of acquiring and transmitting HIV infection and other sexually transmitted infections (STIs) due to their having multiple sexual partners, increased likelihood of unprotected vaginal and anal sexual acts, and greater frequency of abuse of alcohol and other substances [7–9]. In addition, factors such as stigma, harassment, violence, marginalization, limited social support, lower education level, poverty, and mobility may impact HIV acquisition or reduce HIV service uptake. [10,11]. In South Sudan, as in many other countries, sex work is illegal, and FSW/SEA face risk of arrests, extortion of money in exchange for freedom from incarceration, and confiscation of condoms and antiretroviral drugs, as well as the harassment of outreach workers, which poses serious implications for access to and utilization of HIV prevention, care and treatment services by the FSW/SEA [12–14]. The Eagle Survey was conducted to provide reliable data to guide HIV prevention efforts among the FSW/SEA in South Sudan. Researchers for this study characterize FSW/SEA in Nimule for the first time and describe the prevalence of HIV and syphilis in this population.

## Methods

### Study setting, population, and design

The Eagle Survey was conducted in Nimule, a South Sudanese town on the border of Uganda, from January to February of 2017. Respondent-driven sampling (RDS) was used to recruit FSW/SEA aged ≥15 years; who spoke English, Juba Arabic, or Kiswahili; received money, goods, or services in exchange for sex in the past six months; and who resided, worked, or socialized in Nimule for the past month. The RDS was identified as the best methodology for recruitment of the FSW/SEA during formative assessment. General RDS methods have been described by Heckathorn and used in in many countries including in United States, Europe, Asia, Latin America and Africa[15–18].

### Recruitment and data collection

At the beginning of the survey, 5 participants, or “seeds,” were recruited by investigators in consultation with the FSW/SEA peer educators and enrolled in the survey. Seeds were purposively selected to be diverse according to their age, neighborhood of residence, and nationality. All of the seeds were considered influential among their peers. In RDS, the survey participants recruit their peers, creating a form of “chain referral sampling.” Each chain referral forms a wave related to a specific seed. For example, seeds completed the interview process and received three coupons they could use to recruit peers (wave1). The recruits of wave one then completed the interview process and recruited wave two, and so on. As the study progressed, two additional seeds were recruited to include under-represented sub-groups, particularly South Sudanese and FSW/SEA, who operated from their home.

Each participant received three coupons to recruit peers, condoms, transport refunds, and compensation for participation (total of 250 South Sudanese Pounds-SSP, approximately $10 United States Dollars-USD). During the second visit, participants received 100 SSP for transportation and 50 SSP for each successful peer enrollment (250 SSP total, approximately $10 USD maximum).

After screening for eligibility, each participant provided verbal (oral) informed consent and underwent a face-to-face computer-assisted interview (Open Data Kit, Washington, US) at Nimule hospital, inside the study site. Interview domains included: demographics, social cohesion, stigma, HIV knowledge, sexual history, uptake of HIV and STI services, sexual and gender-based violence, and history of STIs. The two-item Patient Health Questionnaire (PHQ-2) was used to screen for depression and the Alcohol Use Disorders Identification Test for alcohol disorders [19,20]. The UNAIDS definition of correctly answering three questions and rejecting two myths regarding HIV was used for assessing comprehensive HIV knowledge [21].

Participants were offered pretest counseling before HIV testing. The HIV testing procedure was done according to the national Ministry of Health (MOH) approved testing algorithm of Determine HIV-1/2 (Alere Inc., MA, US) as the first test. Those showing a reaction were confirmed using Uni-Gold (Trinity Biotech, Ireland). Next, all HIV-positive participants had a CD4 count done using the PIMA analyzer (Alere Inc., MA, US). Then, syphilis testing was done using BIOLINE Syphilis 3.0, followed by the Rapid Plasma Reagin (RPR) test. Participants testing HIV positive were offered and initiated antiretroviral treatment (ART) at Nimule hospital, and those testing positive for syphilis received treatment at the same hospital. Using syndromic management of STIs, participants with symptoms suggestive of STIs were treated as appropriate.

### Data analysis

Data were analyzed using RDS Analyst version 0.62 (Los Angeles, CA, US) using Gile’s Successive Sampling Estimator and Statistical Analysis Software (SAS) version 13.2. Diagnostics were conducted to assess the sample’s independence from seeds. Odds ratios (ORs) and 95% confidence intervals (95% CI) were calculated for bivariate comparisons, and variables significant at p<0.1 and those though not significant but plausible, were included in the multivariate model. HIV infection was the primary endpoint of the analysis.

### Ethical approval

The study received ethical approval from the South Sudan MOH Ethical Review Board and was reviewed by CDC Science Integrity Branch and was conducted consistence with applicable federal law and CDC policy (see 45 C.F.R. part 46; 21 C.F.R. part 56). CDC investigators did not interact with human subjects or have access to identifiable data or specimens for research purposes.

## Results and discussion

In total, 409 FSW/SEA were recruited using seven seeds. The longest chain was 12 waves. The median age of FSW/SEA was 28 years (IQR 23–35). Most were from Uganda (61.4%) and South Sudan (36.8%). Over half (53.0%) of FSW/SEA had no formal education, and 67.9% could not read. One in four (24.7%) FSW/SEA were never married, while 71.6% were either separated/divorced or widowed. Only 12.9% of FSW/SEA resided in Nimule for less than one year. Mobility was evident in the Nimule FSW/SEA population. Nearly one-third (32.1%) traveled out of Nimule in the past 12 months to sell sex and 29.1% indicated they were away from home for more than one month in the past six months. More than half (57.8%) of FSW/SEA did not sleep in the same place most nights. For nearly all (98.8%) FSW/SEA, sex work was their main source of income. Approximately 33.0% earned 3000 SSP (approximately 100 USD) or more monthly. According to the PHQ-2, 45.0% of FSW/SEA screened positive for depression, and based on the AUDIT-3 scale, 34.8% of FSW/SEA engaged in harmful drinking behavior. Finally, about 36.1% reported they dried out or smoked their vagina “Table 1”.

**Table 1:** Characteristics of female sex workers/sexually exploited adolescents in Nimule, South Sudan, January-February 2017.

Variable	Total (n = 409)				HIV Positive, Aware (n = 59)				HIV Positive, Unaware (n = 49)				HIV Negative (n = 300)			
	Sample Proportion		Population Proportion		Sample Proportion		Population Proportion		Sample Proportion		Population Proportion		Sample Proportion		Population Proportion	
	No.	%	%	95% CI	No.	%	%	95% CI	No.	%	%	95% CI	No.	%	%	95% CI
Median Age	IQR 28 [23–35]															
Age, years	409				59				49				300			
15–19	39	9.5	11.3	(7.1–15.5)	2	3.4	2.9	(0.0–5.9)	1	2.0	2.9	(0.0–7.6)	35	11.7	13.7	(8.7–18.8)
20–24	95	23.2	22.8	(18.1–27.6)	7	11.9	14.3	(4.1–25.0)	5	10.2	8.1	(0.8–15.5)	83	27.7	26.3	(20.6–31.9)
25–29	104	25.4	25.3	(20.6–30.0)	25	42.4	42.1	(27.8–55.9)	13	26.5	24.3	(13.4–35.4)	66	22.0	22.6	(17.2–28.0)
30–34	63	15.4	15.7	(11.8–19.5)	9	15.3	16.8	(5.4–28.6)	13	26.5	27.0	(14.3–39.5)	41	13.7	14.1	(9.7–18.4)
35–39	58	14.2	14.3	(10.1–18.5)	13	22.0	20.0	(7.2–32.6)	9	18.4	23.7	(10.3–37.3)	36	12.0	12.1	(8.0–16.2)
40+	50	12.2	10.6	(6.7–14.4)	3	5.1	4.0	(0.3–7.7)	8	16.3	13.9	(5.1–22.8)	39	13.0	11.3	(6.7–15.8)
Country of Birth	409				59				49				300			
Uganda	238	58.2	61.4	(47.2–75.7)	10	17.0	22.8	(5.3–39.9)	18	36.7	39.2	(24.4–54.5)	209	69.7	70.9	(62.0–79.9)
South Sudan	165	40.3	36.8	(23.3–50.2)	47	79.7	72.6	(55.4–90.1)	30	61.2	60.2	(44.9–75.0)	88	29.3	27.6	(19.2–36.0)
Other^†^	6	1.5	1.8	(0.2–3.5)	2	3.4	4.7	(0.0–10.1)	1	2.0	0.6	(0.0–1.5)	3	1.0	1.5	(0.0–3.2)
Literacy	409				59				49				300			
Can Read	141	34.5	32.1	(26.6–37.6)	21	35.6	32.5	(18.2–46.7)	13	26.5	26.2	(13.7–38.9)	107	35.7	32.9	(26.3–39.4)
Cannot Read	268	65.5	67.9	(62.4–73.4)	38	64.4	67.6	(53.3–81.8)	36	73.5	73.8	(61.1–86.3)	193	64.3	67.1	(60.6–73.7)
Highest Education Level	409				59				49				300			
None	207	50.6	53.0	(47.2–58.8)	25	42.4	49.0	(35.4–62.4)	32	65.3	67.3	(54.7–80.6)	149	49.7	51.7	(44.9–58.6)
Primary	128	31.3	31.8	(26.9–36.8)	24	40.7	37.0	(24.7–49.5)	10	20.4	17.7	(7.5–27.3)	94	31.3	32.9	(27.0–38.7)
Secondary	65	15.9	13.6	(9.8–17.3)	8	13.6	13.2	(4.3–22.0)	6	12.2	14.4	(3.6–25.1)	51	17.0	13.6	(8.6–18.5)
Higher	9	2.2	1.6	(0.4–2.7)	2	3.4	0.9	(0.1–1.6)	1	2.0	0.6	(0.0–1.4)	6	2.0	1.8	(0.2–3.5)
Current Marital Status	409				59				49				300			
Single, Never Married	95	23.2	24.7	(19.0–30.5)	10	17.0	20.6	(9.9–31.3)	7	14.3	11.5	(2.9–19.8)	77	25.7	27.0	(20.3–33.8)
Married	14	3.4	3.7	(1.6–5.8)	4	6.8	7.8	(0.4–15.1)	3	6.1	4.5	(0.0–9.8)	7	2.3	2.9	(0.6–5.2)
Separated/Divorced	192	46.9	45.6	(39.9–51.3)	28	47.5	44.8	(30.1–59.6)	23	47.0	47.8	(32.3–62.6)	141	47.0	45.5	(38.7–52.4)
Widowed	108	26.4	26.0	(20.4–31.6)	17	28.8	26.8	(11.9–41.8)	16	32.7	36.3	(21.4–52.2)	75	25.0	24.5	(18.4–30.6)
Monthly Income, SSP	409				59				49				300			
<1,000	71	17.4	17.3	(12.8–21.7)	7	11.9	6.8	(1.7–11.8)	4	8.2	6.7	(0.0–13.5)	60	20.0	20.5	(14.9–26.2)
1,000–1,999	106	25.9	26.6	(21.4–31.7)	12	20.3	20.3	(8.5–32.0)	16	32.7	30.2	(16.6–43.5)	77	25.7	27.0	(20.8–33.3)
2,000–2,999	98	24.0	23.2	(18.6–27.8)	14	23.7	32.2	(18.3–46.2)	15	30.6	30.4	(17.3–44.1)	69	23.0	20.6	(15.3–25.9)
3,000+	134	32.8	33.0	(27.6–38.5)	26	44.1	40.6	(26.5–55.1)	14	28.6	32.8	(19.1–46.3)	94	31.3	31.9	(25.7–38.0)
Sex Work Main Source of Income	409				59				49				300			
Yes	404	98.8	98.8	(97.7–99.9)	57	96.6	97.6	(94.1–101.2)	49	100.0	--	--	297	99.0	98.9	(97.6–100.2)
No	5	1.2	1.2	(0.1–2.3)	2	3.4	2.4	(0.0–5.9)	0	0.0	--	--	3	1.0	1.1	(0.0–2.4)
Length of Stay in Nimule	409				59				49				300			
<1 year	52	12.7	12.9	(8.6–17.3)	13	22.0	22.1	(9.1–34.9)	10	20.4	23.3	(11.4–35.0)	29	9.7	10.0	(6.0–14.1)
1–4 years	192	46.9	44.9	(39.4–50.5)	38	64.4	61.7	(48.2–75.4)	23	46.9	39.0	(24.2–54.0)	130	43.3	42.7	(36.4–49.0)
5–9 years	89	21.8	24.4	(19.3–29.4)	5	8.5	11.1	(3.1–19.2)	4	8.2	13.4	(0.4–26.5)	80	26.7	28.1	(22.0–34.3)
10+ years	76	18.6	17.8	(12.9–22.7)	3	5.1	5.1	(0.2–10.0)	12	24.5	24.3	(10.8–37.8)	61	20.3	19.2	(13.8–24.6)
Travelled Outside of Nimule to Sell Sex in Last 12 Months	406				59				49				297			
Yes	130	32.0	32.1	(26.6–37.5)	31	52.5	48.7	(34.7–62.9)	15	30.6	29.8	(15.2–44.0)	84	28.3	29.5	(23.2–35.9)
No	276	68.0	68.0	(62.5–73.4)	28	47.5	51.3	(37.1–65.3)	34	69.4	70.2	(56.0–84.8)	213	71.7	70.5	(64.1–76.8)
Away from Home More than 1 Month in the Past 6 Months	408				59				49				299			
Yes	128	31.4	29.1	(24.2–34.0)	19	32.2	29.1	(17.1–41.1)	18	36.7	36.4	(21.7–50.8)	90	30.1	28.0	(22.6–33.5)
No	280	68.6	70.9	(66.0–75.8)	40	67.8	71.0	(59.0–82.9)	31	63.3	63.6	(49.2–78.3)	209	69.9	72.0	(66.5–77.5)
Sleep in the Same Place Most Nights	409				59				49				300			
Yes	176	43.0	42.2	(37.0–47.4)	25	42.4	44.3	(31.3–57.8)	20	40.8	44.1	(28.3–59.7)	130	43.3	41.5	(35.4–47.6)
No	233	57.0	57.8	(52.6–63.0)	34	57.6	55.7	(42.2–68.7)	29	59.2	55.9	(40.3–71.7)	170	56.7	58.5	(52.4–64.6)
Screened Positive for Depression	408				58				49				300			
Yes	174	42.7	45.0	(39.3–50.7)	19	32.8	37.8	(23.9–51.4)	22	44.9	43.7	(28.3–59.7)	133	44.3	46.5	(39.4–52.5)
No	234	57.4	55.0	(49.3–60.7)	39	67.2	62.2	(48.6–76.1)	27	55.1	56.3	(40.3–71.7)	167	55.7	53.5	(46.5–60.6)
Harmful Drinking Behavior	409				59				49				300			
Yes	150	36.7	34.8	(29.1–40.5)	27	45.8	41.9	(26.5–57.1)	19	38.8	37.4	(22.4–52.5)	104	34.7	33.3	(26.8–39.8)
No	259	63.3	65.2	(59.6–70.9)	32	54.2	58.2	(42.9–73.5)	30	61.2	62.6	(47.6–77.6)	196	65.3	66.7	(60.3–73.2)
Dry or Smoke Out Vagina**	407				59				49				298			
Yes	161	39.6	36.1	(31.3–41.0)	25	42.4	39.6	(26.1–52.8)	15	30.6	31.2	(18.3–44.5)	121	40.6	36.3	(30.5–42.1)
No	246	60.4	63.9	(59.0–68.8)	34	57.6	60.5	(47.2–73.9)	34	69.4	68.8	(55.5–81.7)	177	59.4	63.7	(57.9–69.5)

CI, Confidence Interval; IQR, Interquartile Range.

^†^Other includes Kenya and Democratic Republic of the Congo.

** Vaginal drying or smoking is used by women to clean, tighten, dry or warm the vagina to enhance hygiene, health or sex.

The median age of initiation of sex work was 23 years (IQR 18–28). More than half (54.5%) of FSW/SEA first engaged in sex work when they were between 15–24 years of age, with the median time engaged in sex work being three years (IQR 2–5). An estimated 38.1% of FSW/SEA had agents who helped them meet clients. Agents included Boda Boda (passenger motorcycle) drivers, hotel managers, receptionists, porters, and lodge and saloon owners. The majority (88.6%) reported they would not stand up in defense of fellow FSW/SEA.

In the last six months, FSW/SEA in Nimule had a median of 7 (IQR: 2–15) main male sex partners “Table 2”. More than half (53.6%) of FSW/SEA did not use a condom at last vaginal or anal sex with a cash client, and 19.8% had a condom break in the last six months. Most (90.4%) did not use a lubricant during vaginal or anal sex in the last six months, with 73.6% indicating they never heard of it while 14.9% indicated they do not like lubricants. Nearly all (99.2%) FSW/SEA lacked comprehensive knowledge of HIV, although 73.5% believed vaginal sex placed them at risk of HIV if a condom was not used “Table 2”.

**Table 2:** Sexual behaviors and healthcare utilization of female sex workers/sexually exploited adolescents in Nimule, South Sudan, January-February 2017.

Variable	Total (n = 409)				HIV Positive, Aware (n = 59)				HIV Positive, Unaware (n = 49)				HIV Negative (n = 300)			
	Sample Proportion		Population Proportion		Sample Proportion		Population Proportion		Sample Proportion		Population Proportion		Sample Proportion		Population Proportion	
	No.	%	%	95% CI	No.	%	%	95% CI	No.	%	%	95% CI	No.	%	%	95% CI
Age at first exchange sex, years	391				58				49				283			
15–19	110	28.1	29.8	(23.9, 35.8)	8	13.8	14.8	(4.3, 25.4)	6	12.2	9.8	(0.8, 18.7)	95	33.6	35.1	(28.1, 42.2)
20–24	104	26.6	24.7	(20.2, 29.2)	16	27.6	27.7	(15.9, 39.8)	13	26.5	24.0	(13.1, 34.6)	75	26.5	24.3	(18.9, 29.7)
25–29	87	22.3	23.3	(18.5, 28.2)	21	36.2	39.3	(25.7, 52.5)	15	30.6	28.7	(15.2, 42.5)	51	18.0	19.8	(14.5, 25.2)
30+	90	23.0	22.1	(17.3, 26.9)	13	22.4	18.2	(7.8, 28.6)	15	30.6	37.5	(23.1, 52.1)	62	21.9	20.7	(15.2, 26.2)
Time engaged in sex work	408				59				49				299			
Median (IQR)	3 (2–5)															
<1 year	14	3.4	5.7	(2.4, 9.0)	0	0.0	--	--	2	4.1	6.2	(0.0, 16.3)	12	4.0	6.6	(2.4, 10.8)
1–2 years	105	25.7	27.4	(22.3, 32.6)	20	33.9	37.7	(22.9, 52.5)	8	16.3	18.3	(6.7, 30.1)	76	25.4	26.7	(20.9, 32.5)
3–4 years	126	30.9	29.8	(24.6, 35.1)	16	27.1	24.1	(11.5, 36.7)	16	32.7	31.2	(17.1, 45.4)	94	31.4	30.7	(24.9, 36.6)
5+ years	163	40.0	37.1	(31.9, 42.2)	23	39.0	38.2	(24.9, 51.4)	23	46.9	44.2	(29.1, 59.4)	117	39.1	36.0	(29.8, 42.2)
Have agent^†^ that helps meet clients	409				59				49				300			
Yes	164	40.1	38.1	(32.8, 43.3)	23	39.0	33.7	(21.7, 45.8)	13	26.5	21.5	(11.0, 32.4)	128	42.7	41.0	(34.6, 47.5)
No	245	59.9	61.9	(56.7, 67.2)	36	61.0	66.4	(54.2, 78.3)	36	73.5	78.5	(67.6, 89.0)	172	57.3	59.0	(52.6, 65.5)
Social cohesion with other female sex workers^‡^	409				59				49				300			
Yes	48	11.7	11.5	(7.7, 15.2)	12	20.3	18.3	(8.9, 27.5)	5	10.2	8.4	(1.7, 15.0)	31	10.3	10.7	(6.5, 14.9)
No	361	88.3	88.6	(84.8, 92.3)	47	79.7	81.8	(72.5, 91.1)	44	89.8	91.6	(85.0, 98.3)	269	89.7	89.3	(85.2, 93.5)
Condom used at last sex act with cash client	388				57				45				285			
Yes	178	45.9	46.4	(38.8, 54.1)	44	77.2	78.6	(63.5, 93.7)	24	53.3	51.6	(34.9, 68.0)	110	38.6	40.2	(33.2, 47.4)
No	210	54.1	53.6	(46.0, 61.2)	13	22.8	21.4	(6.3, 36.5)	21	46.7	48.4	(32.0, 65.1)	175	61.4	59.8	(52.6, 66.8)
Condom used at last vaginal or anal sex act	407				59				48				299			
Yes	253	62.2	59.9	(53.6, 66.2)	48	81.4	76.1	(59.9, 92.5)	30	62.5	62.8	(48.5, 78.2)	174	58.2	56.6	(49.7, 63.5)
No	154	37.8	40.1	(33.8, 46.4)	11	18.6	23.9	(7.5, 40.1)	18	37.5	37.2	(21.9, 51.5)	125	41.8	43.4	(36.5, 50.3)
Had a condom break during vaginal or anal sex in the last 6 months	401				59				49				292			
Yes	90	22.4	19.8	(15.4, 24.1)	26	44.1	41.9	(27.7, 55.8)	6	12.2	10.1	(2.7, 17.2)	58	19.9	17.2	(12.5, 21.9)
No	311	77.6	80.2	(75.9, 84.6)	33	55.9	58.1	(44.2, 72.3)	43	87.8	89.9	(82.8, 97.3)	234	80.1	82.8	(78.1, 87.5)
Used lubricant during anal or vaginal sex in last 6 months	405				59				49				296			
Yes	43	10.6	9.6	(5.3, 14.0)	17	28.8	26.1	(13.0, 39.2)	6	12.2	11.5	(3.1, 19.9)	20	6.8	6.5	(3.2, 9.8)
No	362	89.4	90.4	(86.0, 94.7)	42	71.2	73.9	(60.8, 87.0)	43	87.8	88.5	(80.1, 96.9)	276	93.2	93.5	(90.2, 96.8)
Reason for not using lubricants	351				42				40				268			
Never heard of it	257	73.2	73.6	(68.1, 79.1)	21	50.0	55.4	(38.7, 72.5)	23	57.5	56.5	(39.6, 73.5)	212	79.1	78.2	(72.8, 83.7)
Do not like lubricants	51	14.5	14.9	(10.7, 19.1)	8	19.1	13.1	(1.5, 24.0)	12	30.0	32.6	(16.8, 48.2)	31	11.6	13.0	(8.5, 17.5)
Cannot get them easily/ too expensive	28	8.0	7.2	(4.3, 10.1)	8	19.1	17.4	(6.5, 28.1)	4	10.0	8.0	(0.0, 17.6)	16	6.0	5.7	(2.8, 8.6)
Other^¶^	15	4.3	4.3	(1.7, 6.9)	5	11.9	14.2	(0.4, 28.3)	1	2.5	2.8	(0.0, 7.7)	9	3.4	3.1	(0.8, 5.4)
Comprehensive knowledge of HIV**	408				59				49				299			
Knowledgeable	4	1.0	0.8	(0.0, 1.9)	2	3.4	4.6	(0.0, 15.7)	0	0.0	--	--	2	0.7	0.2	(0.0, 0.5)
Not knowledgeable	404	99.0	99.2	(98.1, 100.0)	57	96.6	95.4	(84.3, 100.0)	49	100.0	--	--	297	99.3	99.8	(99.5, 100.0)
Answer to "Kind of sex that puts one most at risk if condom is not used"	408				59				49				299			
Oral sex	14	3.4	3.1	(1.3, 4.9)	2	4.1	2.5	(0.0, 5.8)	2	4.1	4.0	(0.0, 8.2)	10	3.3	3.1	(0.9, 5.3)
Vaginal sex	302	74.0	73.5	(68.8, 78.1)	51	86.4	87.8	(80.6, 95.0)	37	75.5	79.5	(69.0, 89.8)	213	71.2	70.1	(64.4, 75.8)
Anal sex	19	4.7	4.6	(2.4, 6.7)	3	5.1	5.3	(0.3, 10.4)	0	0.0	--	--	16	5.4	5.0	(2.4, 7.7)
All of the above equally	17	4.2	4.0	(2.0, 6.0)	2	3.4	2.6	(0.0, 5.8)	3	6.1	5.3	(0.0, 11.1)	12	4.0	4.1	(1.6, 6.5)
Don’t know	56	13.7	14.9	(10.8, 18.9)	1	1.7	1.8	(0.0, 5.2)	7	14.3	11.2	(3.1, 19.5)	48	16.1	17.7	(12.7, 22.6)
Agree with statement "you are not as careful about HIV and sex now because there is better treatment for HIV"	383				57				45				280			
Agree	90	23.5	23.7	(19.0, 28.6)	10	17.5	21.2	(11.2, 31.2)	11	24.4	23.4	(8.0, 38.6)	69	24.6	24.3	(18.7, 30.0)
Disagree	293	76.5	76.3	(71.5, 81.0)	47	82.5	78.8	(68.8, 88.8)	34	75.6	76.6	(61.4, 92.0)	211	75.4	75.7	(70.1, 81.3)
Country in which HIV outreach services were accessed	105				30				12				63			
South Sudan	63	60.0	63.7	(51.6, 76.3)	13	43.3	49.1	(24.2, 75.2)	6	50.0	41.7	(9.1, 70.8)	44	69.8	71.7	(57.9, 85.7)
Uganda	39	37.1	33.2	(21.5, 44.5)	16	53.3	49.1	(24.0, 73.1)	6	50.0	58.3	(29.2, 90.9)	17	27.0	24.4	(10.7, 37.9)
Both	3	2.9	3.1	(0.0, 8.1)	1	3.3	1.8	(0.0, 6.1)	0	0.0	--	--	2	3.2	3.9	(0.0, 8.0)
Last time peer educator or outreach worker talked to about HIV	106				30				12				64			
In last 30 days	53	50.0	48.5	(35.9, 61.0)	14	46.7	45.1	(22.5, 67.1)	5	41.7	42.2	(10.7, 73.5)	34	53.1	50.6	(35.5, 65.6)
In the last 1–3 months	18	17.0	19.7	(10.9, 28.8)	7	23.3	26.0	(8.2, 44.1)	2	16.7	26.9	(0.0, 61.4)	9	14.1	16.4	(5.1, 27.9)
In the last 3month-1 year	19	17.9	17.0	(8.6, 25.1)	5	16.7	18.3	(4.7, 32.2)	3	25.0	13.4	(0.0, 27.2)	11	17.2	16.9	(6.7, 27.0)
More than a year ago	16	15.1	14.9	(7.0, 22.7)	4	13.3	10.7	(0.0, 22.5)	2	16.7	17.5	(0.0, 41.4)	10	15.6	16.1	(6.0, 26.2)
Felt the need to hide sex work when seeking healthcare	378				58				47				272			
Yes	51	13.5	14.3	(10.6, 18.0)	8	13.8	17.3	(7.9, 26.9)	8	17.0	18.8	(7.1, 30.7)	35	12.9	13.1	(8.8, 17.5)
No	327	86.5	85.7	(82.0, 89.4)	50	86.2	82.7	(73.1, 92.1)	39	83.0	81.2	(69.3, 92.9)	237	87.1	86.9	(82.5, 91.2)
Experienced STI symptoms^††^ in last 12 months	408				59				49				299			
Yes	58	14.2	13.5	(10.0, 17.1)	10	17.0	16.3	(6.3, 26.3)	8	16.3	17.2	(6.4, 28.1)	40	13.4	12.6	(8.6, 16.5)
No	350	85.8	86.5	(82.9, 90.0)	49	83.1	83.7	(73.7, 93.7)	41	83.7	82.8	(71.9, 93.6)	259	86.6	87.5	(83.5, 91.4)
Went to pharmacy to get treatment for STI symptoms	58				10				8				40			
Yes	34	58.6	57.0	(43.3, 70.5)	6	60.0	57.6	(37.7, 76.1)	5	62.5	69.8	(37.6, 100.0)	23	57.5	54.5	(22.9, 84.7)
No	24	41.4	43.0	(29.5, 56.7)	4	40.0	42.4	(23.9, 62.3)	3	37.5	30.2	(0.0, 62.4)	17	42.5	45.5	(15.3, 77.1)
Ever tested for HIV	408				59				49				299			
Yes	233	57.1	55.3	(48.7, 61.9)	59	100.0	--	--	18	36.7	33.7	(20.9, 46.5)	156	52.2	50.5	(43.1, 58.0)
No	175	42.9	44.7	(38.1, 51.3)	0	0.0	--	--	31	63.3	66.3	(53.5, 79.1)	143	47.8	49.5	(42.0, 56.9)
Country last HIV test was performed in	233				59				18				156			
South Sudan	149	63.9	66.7	(58.3, 75.3)	27	45.8	52.3	(37.5, 66.7)	8	44.4	38.3	(14.1, 61.8)	114	73.1	74.2	(65.1, 83.4)
Uganda	84	36.1	33.4	(24.7, 41.8)	32	54.2	47.8	(33.4, 62.5)	10	55.6	61.7	(38.2, 85.9)	42	26.9	25.8	(16.6, 34.9)
Time since last HIV test	224				56				18				150			
<6 months	97	43.3	45.6	(38.7, 52.9)	17	30.4	37.8	(22.4, 53.3)	6	33.3	39.2	(14.0, 65.7)	74	49.3	48.8	(40.4, 57.2)
7–12 months	53	23.7	24.3	(17.6, 31.2)	9	16.1	15.3	(4.9, 25.7)	6	33.3	31.3	(8.6, 53.9)	38	25.3	26.7	(18.4, 35.0)
1–2 years	40	17.9	14.2	(9.4, 18.6)	13	23.2	17.3	(5.8, 28.8)	4	22.2	22.4	(0.1, 44.8)	23	15.3	12.4	(6.8, 17.8)
3+ years	34	15.2	15.8	(10.1, 21.7)	17	30.4	29.7	(14.3, 44.9)	2	11.1	7.1	(0.0, 17.4)	15	10.0	12.1	(6.5, 17.9)
CD4 count	79				45				33				1			
<200	7	8.9	9.1	(1.1, 17.2)	4	8.9	11.7	(0.8, 22.5)	3	9.1	6.1	(0.0, 12.1)	0	0.0	--	--
200–349	8	10.1	8.4	(2.9, 13.7)	4	8.9	8.4	(1.4, 15.3)	4	12.1	9.2	(0.0, 18.5)	0	0.0	--	--
350–499	20	25.3	23.0	(12.6, 32.7)	11	24.4	18.1	(7.8, 28.4)	9	27.3	31.5	(13.0, 50.8)	0	0.0	--	--
500+	44	55.7	59.5	(48.7, 71.2)	26	57.8	61.9	(47.8, 76.0)	17	51.5	53.2	(34.2, 71.8)	1	100.0	--	--
Ever Infected with Syphilis	408				59				48				300			
Yes	44	10.8	10.1	(7.2, 13.0)	20	33.9	33.6	(20.6, 46.8)	8	16.7	16.7	(5.7, 27.4)	16	5.3	5.2	(2.8, 7.7)
No	364	89.2	89.9	(87.0, 92.8)	39	66.1	66.4	(53.2, 79.5)	40	83.3	83.4	(72.6, 94.3)	284	94.7	94.8	(92.3, 97.2)
Active Syphilis Infection***	42				20				8				14			
Yes	40	95.2	94.7	(83.9, 100.0)	18	90.0	88.6	(69.2, 100.0)	8	100.0	--	--	14	100.0	--	--
No	2	4.8	5.3	(0.0, 16.1)	2	10.0	11.4	(0.0, 30.8)	0	0.0	--	--	0	0.0	--	--

^†^Agents includes Boda Boda driver, hotel manager, reception person at hotel, hotel porter, lodge owner, saloon owner, another sex worker, family member, friend, and other.

^‡^Social cohesion includes negotiated with or stood up against police, a madam/broker/pimp and any clients/any other sexual partner in order to help a fellow FSW in past 12 months.

^§^Multiple reasons allowed.

^||^Other includes when I am drunk or high, when I cannot afford to buy a condom when I am afraid to ask my partner to use a condom, when having sex with a non-regular partner, when the person does not ejaculate inside me, and other.

^¶^Other includes partner does not like them, I’m ashamed/embarrassed to buy it because it is associated with homosexuals and others.

**Comprehensive knowledge of HIV is indicated by correctly answering all five questions per the UNAIDS definition.

^††^STI symptoms include abnormal discharge from the vagina and an ulcer or sore on or near vagina.

*** Testing positive on both screening (SD Bioline) and confirmatory test (Rapid plasmin Reagin).

FSW/SEA in Nimule had limited access to HIV prevention services, with 48.5% having talked to the peer educator or outreach worker about HIV in the last 30 days. Only 19.7% indicated talking with a peer educator or outreach worker about HIV between 31–90 days prior. Nearly half of FSW/SEA (44.7%) were never tested for HIV. The most common reason for never testing was not knowing where to test (50.0%). For those tested before, 66.7% were tested in South Sudan, and 45.7% were tested in the last six months.

Thirty-five percent of FSW/SEA did not know where male condoms could be obtained. Only one-third of FSW/SEA (33.3%) received free condoms in the past 12 months, with community health workers being the source of free condoms for 32.7% of those FSW/SEA. Only 13.5% reported experiencing STI symptoms in the last 12 months and 57.0% sought treatment. Estimated HIV prevalence among FSW/SEA in Nimule was 24% (95% CI; 19.4–28.5), and 44% of those with HIV were unaware of their status. Forty-one percent of HIV-infected FSW/SEA had a CD4 count below 500 cells/mm^3^. Ten percent of FSW/SEA were ever infected with syphilis, while 9.2% (95% CI; 6.5–11.9) had active syphilis “Table 2” above.

In bivariate analysis, FSW/SEA ages 15–19 years were less likely to be living with HIV compared to those age 20 years and above “Table 3”. Being non-South Sudanese, staying <1 year in Nimule compared to a year or more, condom use during their last sex act with cash clients, previously testing for HIV, previous HIV test in Uganda, and having active syphilis were associated with HIV infection (p<0.001). In multivariable analysis, those with active syphilis were seven times as likely to have HIV (95% CI: 2.2–21.9).

**Table 3:** Correlates of HIV infection among female sex workers /sexually exploited adolescents in Nimule, South Sudan, January-February 2017.

Variable	Prevalence		Bivariate Models			Multivariate Model		
	%	95% CI	OR	95% CI	p-value	AOR	95% CI	p-value
Age, years								
15–19	7.9	(0.0, 16.5)	0.25	(0.0, 0.5)	<0.001	0.1	(0.0, 0.8)	0.01
20–24	12.6	(6.0, 19.3)	0.3	(0.1, 0.2)		0.3	(0.1, 0.8)	
25–29	36.5	(27.3, 45.8)	1.0	Ref		1.0	Ref	
30–34	34.9	(23.2, 46.7)	0.9	(0.9, 1.8)		1.4	(0.5, 4.2)	
35–39	37.9	(25.4, 50.4)	1.1	(0.6, 2.1)		2.2	(0.7, 6.8)	
40+	22.0	(10.5, 33.5)	0.5	(0.2, 1.1)		1.0	(0.2, 4.0)	
Country of Birth								
South Sudan	11.8	(7.7, 15.9)	1.0	Ref	<0.001	1.0	Ref	0.02
Uganda	46.7	(39.1, 54.3)	6.5	(4.0, 10.8)		5.9	(1.5, 22.6)	
Other^†^	50.0	(10.0, 90.0)	7.5	(1.4, 38.8)		26.5	(0.7, —)	
Literacy								
Can Read	24.1	(17.1, 31.2)	0.8	(0.5, 1.3)	0.43			
Cannot Read	27.7	(22.4, 33.1)	1.0	Ref				
Marital Status								
Single, Never Married	18.1	(10.3, 25.9)	1.0	Ref	0.02	1.0	Ref	0.40
Married	50.0	(23.8, 76.2)	4.5	(1.4, 14.6)		2.0	(0.3, 13.3)	
Separated, Divorced, or Widowed	28.0	(22.9, 33.18)	1.8	(1.0, 3.2)		0.7	(0.2, 2.0)	
Monthly Income, SSP								
<1,000	15.5	(7.1, 23.9)	1.0	Ref	0.10	1.0	Ref	0.15
1,000–1,999	26.7	(18.2, 35.1)	2.0	(0.9, 4.3)		1.1	(0.3, 4.0)	
2,000–2,999	29.6	(20.6, 38.6)	2.3	(1.1, 5.0)		0.6	(0.2, 2.3)	
3,000+	29.9	(22.1, 37.6)	2.3	(1.1, 4.9)		0.3	(0.1, 1.2)	
Sex Work Main Source of Income (versus no)	26.3	(22.0, 30.6)	0.5	(0.1, 3.3)	0.51			
Length of Stay in Nimule								
<1 year	44.2	(30.7, 57.7)	1.0	Ref	<0.001	1.0	Ref	0.35
1–4 years	31.9	(25.3, 38.5)	0.6	(0.3, 1.1)		0.8	(0.3, 2.0)	
5–9 years	10.1	(3.9, 16.4)	0.1	(0.1, 0.3)		0.2	(0.1, 1.2)	
10+ years	19.74	(10.8, 28.7)	0.3	(0.1, 0.7)		0.5	(0.1, 3.0)	
Traveled Outside of Nimule to Sell Sex in Last 12 Months (versus no)	35.4	(27.2, 43.6)	1.9	(1.2, 23.0)	0.01	0.9	(0.4, 2.0)	0.77
Away From Home More Than 1 Month in the Past 6 Months (versus no)	29.1	(21.2, 37.0)	1.2	(0.8, 1.9)	0.43			
Sleep in the Same Place Most Nights (versus no)	25.7	(19.2, 32.2)	0.9	(0.6, 1.5)	0.76			
Screened Positive for Depression (versus no)	23.6	(17.3, 29.9)	0.8	(0.5, 1.2)	0.28			
Harmful Drinking Behavior (versus no)	30.7	(23.39, 38.1)	1.4	(0.9, 2.2)	0.15			
Dry or Smoke Out Vagina (versus no)	24.8	(18.2, 31.5)	0.9	(0.6, 1.4)	0.52			
Time engaged in sex work								
<1 year	14.3	(0.0, 32.6)	0.4	(0.1, 2.0)	0.67			
1–2 years	26.9	(18.4, 35.5)	0.9	(0.5, 1.)				
3–4 years	25.4	(17.8, 33.0)	0.9	(0.5, 1.5)				
5+ years	28.2	(21.3, 35.1)	1.0	Ref				
Have agent^‡^ that helps meet clients (versus no)	22.0	(15.6, 28.3)	0.7	(0.4, 1.1)	0.09	0.9	(0.4, 1.9)	0.79
Social cohesion with other female sex workers^§^ (versus no)	35.4	(21.9, 49.0)	1.6	(0.9, 3.1)	0.15			
Condom used at last sex act with cash client (versus no)	38.2	(31.1, 45.3)	3.2	(2.0, 5.1)	<0.001	2.2	(0.9, 5.7)	0.09
Condom used at last vaginal or anal sex act (versus no)	31.0	(25.2, 36.7)	1.9	(1.2, 3.1)	0.01	0.6	(0.2, 2.0)	0.41
Had a condom break during vaginal or anal sex in the last 6 months (versus no)	35.6	(25.7, 45.5)	1.7	(1.0, 2.8)	0.04	0.8	(0.3, 1.7)	0.52
Comprehensive knowledge of HIV^||^ (versus not knowledgeable)	50.0	(1.0, 99.0)	2.8	(0.4, 20.1)	0.32			
Last time peer educator or outreach worker talked to about HIV								
In last 3 months	39.4	(28.1, 50.8)	2.3	(1.3, 4.0)	0.01	1.9	(0.8, 4.4)	0.41
In the last 1 year	42.1	(19.9, 64.3)	2.6	(1.0, 6.7)		0.7	(0.1, 3.1)	
More than 1 year ago	37.5	(13.8, 61.2)	2.1	(0.8, 6.1)		1.7	(0.4, 7.6)	
Never	21.9	(17.2, 26.6)	1.0	Ref		1.0	Ref	
Felt the need to hide sex work when seeking healthcare (versus no)	31.4	(18.6, 44.1)	1.2	(0.6, 2.3)	0.55			
Experienced STI symptoms^¶^ in last 12 months (versus no)	31.0	(19.1, 42.9)	1.3	(0.7, 2.4)	0.41			
Went to pharmacy to get treatment for STI symptoms (versus no)	32.4	(16.634, 48.1)	1.6	(0.4, 3.6)	0.80			
Ever tested for HIV (versus no)	33.1	(27.0, 39.1)	2.3	(1.4, 3.7)	<0.001			
Country last HIV test was performed in								
South Sudan	23.5	(16.7, 30.3)	1.0	Ref	<0.001	1.0	Ref	0.27
Uganda	50.0	(39.3, 60.7)	3.2	(1.8, 5.8)		1.6	(0.7, 4.0)	
Active Syphilis Infection (versus no)	65.0	(50.2, 79.8)	6.5	(3.3, 13.1)	<0.001	7.0	(2.2, 21.9)	<0.001

†Other includes Kenya and Democratic Republic of the Congo.

‡Agents includes Boda Boda driver, hotel manager, reception person at hotel, hotel porter, lodge owner, saloon owner, another sex worker, family member, friend, and other.

§Social cohesion includes negotiated with or stood up against police, a madam/broker/pimp, and any clients/any other sexual partner to help a fellow sex worker in the past 12 months.

||Comprehensive knowledge of HIV is indicated by correctly answering all five questions per the UNAIDS definition.

¶STI symptoms include abnormal discharge from the vagina and an ulcer or sore on or near vagina.

This survey, the first to estimate HIV and syphilis prevalence in this border town which forms the land border between South Sudan and Uganda, reveals a high prevalence of HIV and syphilis among FSW/SEA. The HIV prevalence among the FSW/SEA was eight times higher than the general population [2]. This survey’s findings also reveal the importance of integrating HIV and STI services for key populations given that FSW/SEA with active syphilis are seven times as likely as those without to have HIV. It This study also highlights the potential impact of coordinating services for FSW/SEA across borders. While most FSW/SEA stayed in Nimule for more than one year, one in three sold sex outside of Nimule in the last six months, reflecting the population’s mobility [22]. Some of these women may have sold sex across the border in Uganda, where HIV prevalence among the general population [23] is higher than in Nimule. In addition, Nimule is a cosmopolitan town. Like other major cross-border towns, Nimule has various ethnic groups and business activities with many truck drivers making stopovers in this border town for clearance. The truck driver stopovers could be a major contributing factor in the increased HIV infection and high prevalence rate [24].

More than half of the sex workers engaged in sex work when they were young and have been in the business for a median of three years and the median number of clients they serviced in the last six months was seven. This increases their exposure to the risk of HIV. Similar findings exist in other sub-Saharan Africa regions [25–27] to Nimule, where more than half of the FSW/SEA did not use condoms during their last sexual encounter or one in every five had a condom break. The prevalence of condom rupture may be connected to FSW/SEA’s limited access to lubricants and the practice of smoking out or drying out the vagina [28]. In addition, nearly all the FSW/SEA in Nimule lacked comprehensive knowledge of HIV. Most FSW/SEA were illiterate and had limited interaction with the outreach workers making it even harder for FSW/SEA to access and utilize condoms and lubricants. This finding is consistent with other studies done elsewhere in Rwanda, Uganda, Brazil, Kenya, Central African Republic [25,29–32].

This study noted the strong correlation between HIV and syphilis through bivariate and multivariate analysis. HIV and syphilis have a similar mode of transmission [25]. However, the painless ulcerative and asymptomatic nature of syphilis among many women increases the risk of HIV transmission, as documented elsewhere in sub-Saharan Africa [25,33–35].

## Conclusions

The HIV and syphilis prevalence is high in Nimule, and syphilis infection is strongly associated with HIV among the FSW/SEA in Nimule. This study underscores the importance of tailored, comprehensive peer-to-peer interventions to integrate the identification of FSW/SEA with HIV and syphilis prevention and control programs. Perhaps the use of recent dual HIV/syphilis diagnostics/tests [36] could be considered to improve identification and prompt linkage of FSW/SEA to care and treatment for both HIV and syphilis in conjunction with HIV combination prevention approaches including comprehensive, tailored HIV information and condom use promotion [37]. In addition, there is a need for structured and well-defined cross-border collaboration with Uganda to ensure the FSW/SEA can access these services on either side of the border.

Access to Habash (Ethiopian and Eritrean) FSW/SEA, who mainly worked in a hotel setting, was difficult due to the language barrier. In addition, the eligibility criteria may not allow these findings to be generalized to FSW/SEA outside of Nimule. Lastly, given mobility of FSW/SEA and the easy movement between residents of Nimule in South Sudan and Elegu in Uganda, the FSW/SEA who could have been in Uganda at the time of this study could not participate.

## Supporting information

S1 File (DOCX)

## References

[1] Ministry of Health, Republic of South Sudan. South Sudan Antenatal Clinic Sentinel Surveillance for HIV, Syphilis and Hepatitis B. 2017; Ministry of Health: Juba, South Sudan.

[2] UNAIDS. South Sudan | UNAIDS 2018 [cited 2020 June 10]; Available from: http://www.unaids.org/en/regionscountries/countries/southsudan/.

[3] Ministry of Health, Republic of South Sudan. A Bio-Behavioral HIV Survey of Female Sex Workers in Juba, South Sudan: The Eagle Survey. 2016; Ministry of Health: Juba, South Sudan.

[4] Duff P, Birungi J, Dobrer S, Akello M, Muzaaya G, Shannon K. Kate Shannon. Social and structural factors increase inconsistent condom use by sex workers’ one-time and regular clients in Northern Uganda. AIDS Care. 2018;30:6, 751–759. doi: 10.1080/09540121.2017.1394966 29067831

[5] Shannon K, Strathdee SA, Goldenberg SM, Duff P, Mwangi P, Rusakova M et al. Global epidemiology of HIV among female sex workers: influence of structural determinants. Lancet. 2015;January 3; 385(9962): 55–71. doi: 10.1016/S0140-6736(14)60931-4 25059947 PMC4297548

[6] Shannon K, Montaner JGS. The politics and policies of HIV prevention in sex work. Lancet Infect Dis. 2012; July; 12(7): 500–502. doi: 10.1016/S1473-3099(12)70065-8 22424776 PMC3675901

[7] Scorgie F, Chersich MF, Ntaganira I, Gerbase A, Lule F, Lo Y-R. Socio-demographic characteristics and behavioral risk factors of female sex workers in sub-Saharan Africa: a systematic review. AIDS Behav. 2012; 16(4): p. 920–33. doi: 10.1007/s10461-011-9985-z 21750918

[8] Cohen MS. Sexually transmitted diseases enhance HIV transmission: no longer a hypothesis. Lancet. 1998; 351(Suppl 3): p. 5–7. doi: 10.1016/s0140-6736(98)90002-2 9652712

[9] Freeman EE, Weiss HA, Glynn JR, Cross PL, Whitworth JA, Hayes RJ. Herpes simplex virus 2 infection increases HIV acquisition in men and women: systematic review and meta-analysis of longitudinal studies. AIDS. 2006; 20(1): p. 73–83. doi: 10.1097/01.aids.0000198081.09337.a7 16327322

[10] Udoh IA, Mantell JE, Sandfort T, Eighny MA. Potential pathways to HIV/AIDS transmission in the Niger Delta of Nigeria: poverty, migration and commercial sex. AIDS Care. 2009; 21(5): p. 567–74. doi: 10.1080/09540120802301840 19444664 PMC2879970

[11] Hargreaves JR, Busza J, Mashati P, Fearon E, Cowan FM. Overlapping HIV and sex-work stigma among female sex workers recruited to 14 respondent-driven sampling surveys across Zimbabwe, 2013. AIDS Care. 2017; 29(6): p. 675–685. doi: 10.1080/09540121.2016.1268673 27998178

[12] United States Department of State. Trafficking in Persons Report—South Sudan, 28 June 2018. Available from: https://www.refworld.org/docid/5b3e0a7da.html [accessed 28 April 2020].

[13] Simi M, Rhodes T. Violence, dignity and HIV vulnerability: street sex work in Serbia. Sociol Health Ill. 2009; 31(1) 1–16. doi: 10.1111/j.1467-9566.2008.01112.x 19144087

[14] The Open Society Foundation. How policing practices put sex workers and HIV services at risk in Kenya, Namibia, Russia, South Africa, the United States, and Zimbabwe. 2012. Available from: https://www.opensocietyfoundations.org/uploads/77d576b0-41b0-45d8-ba72-afae15438e50/criminalizing-condoms-20120717.pdf [accessed 11 June 11 2020].

[15] Heckathorn DD. Respondent-driven sampling: A new approach to the study of hidden populations. Soc Probl. 1997; 44: 174–199. doi: 10.2307/3096941

[16] Heckathorn DD. Snowball versus respondent-driven sampling. Sociol Methodol. 2011; 41(1): 355–366. doi: 10.1111/j.1467-9531.2011.01244.x 22228916 PMC3250988

[17] Heckathorn DD. Respondent-driven sampling II: Deriving valid population estimates from chain-referral samples of hidden populations. Soc Probl. 2002; Volume 49, Issue 1, 1 February 2002: 11–34. doi: 10.1525/sp.2002.49.1.11

[18] Malekinejad M, Johnston LG, Kendall C, Kerr LRFS, Rifkin MR, Rutherford GW. Using respondent-driven sampling methodology for HIV biological and behavioral surveillance in international settings: a systematic review. AIDS Behav. 2008; 12(4 Suppl): p. S105–30. doi: 10.1007/s10461-008-9421-1 18561018

[19] Kroenke K, Spitzer RL, Williams JBW. The Patient Health Questionnaire-2: validity of a two-item depression screener. Med Care. 2003; 41(11): p. 1284–92. doi: 10.1097/01.MLR.0000093487.78664.3C 14583691

[20] World Health Organization. The Alcohol Use Disorders Identification Test: Guidelines for Use in Primary Care. 2001. Geneva, Switzerland: World Health Organization.

[21] UNAIDS. Global AIDS response progress reporting 2014: construction of core indicators for monitoring the 2011 United Nations political declaration on HIV and AIDS. 2014. Geneva, Switzerland: UNAIDS.

[22] Bershteyn A, Mutai KK, Akullian AN, Klein DJ, Jewell BL, Mwalili SM., The influence of mobility among high-risk populations on HIV transmission in Western Kenya. Infectious Disease Modelling. 2018; 3 97e106 doi: 10.1016/j.idm.2018.04.001 30839863 PMC6326217

[23] Hladik W, Baughman AL, Serwadda D, Tappero JW, Kwezi R, Nakato ND, et al. Burden and characteristics of HIV infection among female sex workers in Kampala, Uganda–a respondent-driven sampling survey. BMC Public Health. 2017;17:565. doi: 10.1186/s12889-017-4428-z 28601086 PMC5466716

[24] IOM Kenya. Response Analysis: Combination HIV prevention Programming along the Northern Transport Corridor in Kenya. 2011. Available from: http://kenya.iom.int/sites/default/files/Response_Analysis_along_the_Transport_Corridors_in_Kenya.pdf [accessed 4 June 2020].

[25] Mutagoma M, Nyirazinyoye L, Sebuhoro D, Riedel DJ, Ntaganira J. Syphilis and HIV prevalence and associated factors to their co-infection, hepatitis B and hepatitis C viruses prevalence among female sex workers in Rwanda. BMC Infect Dis. 2017; 17:525. doi: 10.1186/s12879-017-2625-0 28754104 PMC5534065

[26] Augusto Ângelo do Rosário, Young Peter W., Horth Roberta Z., Celso Inguane, Sathane Isabel, Ngale Katia, et al. High burden of HIV infection and risk behaviors among female sex workers in three main urban areas of Mozambique. AIDS Behav. 2016; April; 20(4): 799–810. doi: 10.1007/s10461-015-1140-9 26238035 PMC5092171

[27] Morris CN, Ferguson AG. Estimation of the sexual transmission of HIV in Kenya and Uganda on the trans-Africa highway: the continuing role for prevention in high risk groups. Sex Transm Infect. 2006;82:368–371. doi: 10.1136/sti.2006.020933 16854995 PMC2563851

[28] Low N, Chersich MF, Schmidlin K, Egger M, Francis SC, van de Wijgert JHHM et al. Intravaginal practices, bacterial vaginosis, and HIV infection in women: individual participant data meta-analysis. PLoS Med. 2011;8(2):e1000416. doi: 10.1371/journal.pmed.1000416 21358808 PMC3039685

[29] Szwarcwald Célia Landmann, Damacena Giseli Nogueira, de Souza-Júnior Paulo Roberto Borges, Guimarães Mark Drew Crosland, da Silva de Almeida Wanessa, de Souza Ferreira Arthur Pate et al. Factors associated with HIV infection among female sex workers in Brazil. Medicine. 2018; 97:S1 doi: 10.1097/MD.0000000000009013 29912814 PMC5991538

[30] Bhattacharjee Parinita, Helgar K Musyoki Marissa Becker, Musimbi Janet, Kaosa Shem, Kioko Japheth, et al. HIV prevention programme cascades: insights from HIV programme monitoring for female sex workers in Kenya. J Int AIDS Soc. 2019; 22(S4):e25311. doi: 10.1002/jia2.25311 31328436 PMC6643069

[31] Longo JDD, Simaleko MM, Diemer HS-C, Gresenguet G, Brucker G, Belec L. Risk factors for HIV infection among female sex workers in Bangui, Central African Republic. PLoS ONE 2017; 12(11): e0187654. doi: 10.1371/journal.pone.0187654 29108022 PMC5673229

[32] Bukenya J, Vandepitte J, Kwikiriza M, Weiss HA, Hayes R, Grosskurth H. Condom use among female sex workers in Uganda. AIDS Care. 2013; 25:6, 767–774. doi: 10.1080/09540121.2012.748863 23216336

[33] Ouedraogo HG, Meda IB, Zongo I, Ky-Zerbo O, Grosso A, Samadoulougou BC et al. Syphilis among female sex workers: Results of point-of-care screening during a cross-sectional behavioral survey in Burkina Faso, West Africa. Int J Microbiol. 2018;Nov 8;2018:4790560. doi: 10.1155/2018/4790560 30532783 PMC6250000

[34] Niama FR, Bongolo NCL, Meyengue PI, Mboussou FF, Bayonne ESK, Nzingoula FMK et al. A study on HIV, syphilis, and hepatitis B and C virus infections among female sex workers in the Republic of Congo. Archives of Public Health. 2017;75:21. doi: 10.1186/s13690-017-0189-5 28503303 PMC5421326

[35] Vu L, Misra K. High burden of HIV, syphilis and HSV-2 and factors associated with HIV infection among female sex workers in Tanzania: Implications for early treatment of HIV and pre-exposure prophylaxis (PrEP). AIDS and Behav. 2018;22: 1113–1121. doi: 10.1007/s10461-017-1992-2 29159593

[36] World Health Organization. WHO information note on the use of dual HIV/Syphilis rapid diagnostic tests (RDT). Available from: http://www.who.int/reproductivehealth/publications/rtis/dual-hiv-syphilisdiagnostic-tests/en/.

[37] Chersich MF, Luchters S, Ntaganira I, Gerbase A, Lo Y-R, Scorgie F, Steen R. Priority interventions to reduce HIV transmission in sex work settings in sub-Saharan Africa and delivery of these services. J Intl AIDS Soc. 2013; 16:17980. doi: 10.7448/IAS.16.1.17980 23462140 PMC3589546

